# A randomized phase 2B trial of vancomycin versus daptomycin for the treatment of methicillin-resistant *Staphylococcus aureus* bacteremia due to isolates with high vancomycin minimum inhibitory concentrations – results of a prematurely terminated study

**DOI:** 10.1186/s13063-018-2702-8

**Published:** 2018-06-01

**Authors:** Shirin Kalimuddin, Yvonne F. Z. Chan, Rachel Phillips, Siew Pei Ong, Sophia Archuleta, David Chien Lye, Thuan Tong Tan, Jenny G. H. Low

**Affiliations:** 1 Department of Infectious Diseases, Singapore General Hospital, 20 College Road, Singapore, 169856 Singapore; 2 School of Population Health and Environmental Sciences, Faculty of Life Sciences and Medicine, King’s College London, Guy’s Campus, London, SE1 1UL UK; 3 NIHR Biomedical Research Centre at Guy’s and St. Thomas’ NHS Foundation Trust and King’s College London, Guy’s Hospital, London, SE1 9RT UK; 4 Geriatric Education and Research Institute, 2 Yishun Central 2, Singapore, 768024 Singapore; 5 Division of Infectious Diseases, National University Health System, 5 Lower Kent Ridge Rd, Singapore, 119074 Singapore; 6 Department of Medicine, Yong Loo Lin School of Medicine, National University of Singapore, 21 Lower Kent Ridge Rd, Singapore, 119077 Singapore; 7 Department of Infectious Diseases, Communicable Disease Centre, Tan Tock Seng Hospital, 11 Jalan Tan Tock Seng, Singapore, 308433 Singapore

**Keywords:** Methicillin-resistant *Staphylococcus aureus*, Vancomycin, Daptomycin, Minimum inhibitory concentration, Bacteremia

## Abstract

**Background:**

Studies have suggested the reduced effectiveness of vancomycin against methicillin-resistant *Staphylococcus aureus* (MRSA) bloodstream infections with high vancomycin minimum inhibitory concentrations. Alternative agents such as daptomycin may be considered. We conducted a randomized controlled study comparing daptomycin against vancomycin in the treatment of MRSA bloodstream infections with high vancomycin minimum inhibitory concentrations.

**Methods:**

Patients were randomized to receive vancomycin or daptomycin for a minimum of 14 days. The primary end point was the rate of all-cause mortality at day 60.

**Results:**

A total of 14 patients were randomized in this study, with 7 patients in each treatment arm. The study was terminated early due to slow patient accrual. At day 60, there was one death in the vancomycin arm and none in the daptomycin arm. The median time to microbiological clearance was 4 days in both arms (IQR 3–5 days in the vancomycin arm and 3–7 days in daptomycin arm). Only one patient in the vancomycin arm had recurrence of bacteremia. Rates of adverse events were similar in both arms. There was one case of musculoskeletal toxicity and one case of drug-related nephrotoxicity - both events occurred in the daptomycin arm. None of the patients in either treatment arm required cessation of study treatment or addition of a second anti-MRSA agent because of worsening infection.

**Conclusion:**

Based on the limited number of patients evaluated in this study, it remains unclear if alternative, more expensive agents such as daptomycin are superior to vancomycin in the treatment of high vancomycin minimum inhibitory concentration MRSA bloodstream infections. More studies are urgently needed but investigators may wish to consider employing novel, alternative trial methodologies to ensure a greater chance of success.

**Trial registration:**

ClinicalTrials.gov, NCT01975662. Registered on 5 November 2013.

## Background

*Staphylococcus aureus* is one of the most common human pathogens that cause a wide range of clinical infections worldwide. Infections caused by methicillin-resistant *S. aureas* (MRSA) are associated with increased mortality and morbidity and increased length and cost of hospitalization [1, 2]. Moreover, because MRSA is multidrug resistant, it leaves clinicians with few effective antimicrobial options [3]. Treating *S*. *aureus* infections has become increasing challenging in recent, with the emergence of highly virulent MRSA strains.

Vancomycin, a tricyclic glycopeptide, is the standard first line treatment for patients with MRSA bloodstream infections (BSI). Consensus guidelines, however, recommend clinicians to consider alternative agents for MRSA infections when the vancomycin minimum inhibitory concentration (MIC) is > 1 μg/ml, especially when clinical failure is suspected with vancomycin treatment [4]. One such alternative agent is daptomycin - a calcium-dependent cyclic lipopeptide that is rapidly bactericidal against many gram-positive organisms including MRSA [5, 6]. A number of recent studies have also suggested that vancomycin use, especially in infections caused by MRSA with high vancomycin MICs, may be associated with poorer clinical outcomes when compared with an alternative anti-MRSA agent such as daptomycin; however, these were retrospective and non- randomized studies [7–10].

To date, there has been no head-to-head randomized trial comparing the safety and efficacy of daptomycin and vancomycin in the treatment of BSIs due to MRSA with high vancomycin MICs. Clinicians are often faced with the dilemma of using the cheaper standard treatment, i.e. vancomycin, or switching to a newer and more expensive alternative such as daptomycin in treating such serious infections. This study was designed as a phase 2B randomized controlled trial to evaluate the efficacy of daptomycin versus vancomycin in reducing all-cause mortality in the treatment of MRSA BSIs due to isolates with high vancomycin MICs.

## Methods

This study was an open-label randomized controlled phase 2B trial conducted in a tertiary hospital in Singapore. The trial was granted ethics approval by the Singhealth Centralized Institute Review Board (CIRB) (approval ID 2013/846/E). The trial was registered on ClinicalTrials.gov (NCT01975662). The study protocol was published in *Trials* [11]. Subjects were first enrolled on 13 February 2014 with the last subject visit on 25 September 2015.

### Study population

Patients were eligible for the study if they were ≥ 21 years of age, were inpatients at the time of enrolment and had a BSI due to MRSA with a vancomycin MIC ≥ 1.5 μg/ml but < 2 μg/ml as determined by the Epsilometer test (E-test) or the VITEK™-2 system (bioMerieux, Marcy l’Etoile, France). Patients were excluded if they were allergic to vancomycin or daptomycin, pregnant or breastfeeding, unable to comply with study treatments and procedures, unable to provide consent or had no legally authorized representatives, currently enrolled in or had participated in an interventional antibiotic or vaccine trial within the past 3 months, expected to have less than 24 h of life expectancy, polymicrobial bacteremia, pneumonia, receiving treatment with linezolid, tigecyline or ceftaroline for more than 96 h prior to enrolment, receiving vancomycin or daptomycin treatment for more than 5 days prior to enrolment, or if they had baseline serum creatine kinase (CK) more than 1.5 times the upper limit of normal, presence of prosthetic heart valves, or any other significant condition that would, in the opinion of the investigator, compromise the patient’s safety or outcome of the trial. The study exclusion criteria were amended from the published protocol [11] on 7 August 2014 after consultation with the trial steering committee in an effort to improve recruitment, without compromising the rigor of the study design. When the trial was first commenced, patients were also excluded if they were on vancomycin or daptomycin for more than 96 h prior to enrolment, were on linezolid, tigecycline or ceftaroline immediately prior to enrolment, had previous blood cultures positive for MRSA in the preceding month, or if there was more than 48 h between enrolment and reporting by the microbiology laboratory of MRSA BSI with vancomycin MIC ≥ 1.5 μg/ml.

### Antimicrobial therapy

Participants were randomized to receive either intravenous daptomycin or vancomycin. Patients in the vancomycin arm received a starting dose of 15 mg/kg body weight of vancomycin infused every 12 h over 2 h with appropriate dose adjustments in those with a creatinine clearance < 50 ml/min, so as to achieve a vancomycin trough level of 15–20 μg/ml. Patients who were on vancomycin prior to study inclusion had their doses adjusted accordingly to achieve a vancomycin trough of 15–20 μg/ml. This dosing regimen was based on a consensus statement of the American Society of Health-System Pharmacists, the IDSA, and The Society of Infectious Diseases Pharmacists on guidelines for vancomycin dosing [12, 13].

Patients randomized to the daptomycin arm with uncomplicated bacteremia received 6 mg/kg body weight of daptomycin infused over 30 min every 24 h. This dosing regimen was based on IDSA guidelines, which recommend a minimum dose of 6 mg/kg of daptomycin every 24 h [4]. Some experts however do recommend a higher dose of 8 mg/kg body weight in complicated infection or treatment failure due to the concentration-dependent effect of daptomycin [14, 15]. Hence, daptomycin was dosed at 8 mg/kg body weight every 24 h in patients with complicated bacteremia or endocarditis. In patients with creatinine clearance < 30 ml/min or in patients on hemodialysis, daptomycin was administered every 48 h as per manufacturer’s guidelines. There are currently no recommended peak or trough levels for daptomycin.

Duration of treatment was determined based on the type of bacteremia. Patients with uncomplicated bacteremia received a minimum of 14 days antibiotics and those with complicated bacteremia or infective endocarditis received a minimum of 28–42 days of antibiotics from the date that microbiological clearance was achieved. Uncomplicated bacteremia was defined as the isolation of MRSA from enrolment blood cultures in patients without endocarditis and without evidence of spread to other organs. Complicated bacteremia without endocarditis was defined as the isolation of MRSA from blood cultures beyond 4 days from initial positive culture, the presence of spread of infection, or infection of prostheses not removed within 4 days. The definition of “definite” or “possibl” endocarditis (complicated bacteremia) was determined using the modified Duke criteria [16].

### Study outcomes

The primary objective of this pilot study was to compare the efficacy of daptomycin versus vancomycin in the treatment of MRSA BSIs due to isolates with high vancomycin MICs (i.e. ≥ 1.5 μg/ml) in terms of all-cause mortality 60 days from the time of index blood culture. Index blood culture was defined as the first blood culture which grew MRSA with a vancomycin MIC ≥ 1.5 μg/ml.

The planned secondary efficacy end points of the study were as follows: (1) to compare the rates of “clinical failure” as per the definitions in studies by (i) Moore et al. [7] i.e. a composite of all-cause mortality 60 days from index blood culture, microbiologic failure (defined as growth of MRSA in blood cultures ≥ 7 days from index blood culture) and/or recurrence of MRSA BSI (defined as a positive blood culture for MRSA at any point in time from the point of microbiological clearance up to 60 days from the index blood culture), (ii) Cheng et al. [8] i.e. a composite of microbiologic failure and/or recurrence of MRSA BSI, and (iii) Murray et al. [9] i.e. a composite of all-cause mortality 60 days from index blood culture and/or microbiologic failure; and (2) to compare time to microbiological clearance (defined as two consecutive MRSA-negative blood cultures). However, due to the small number of patients eventually recruited, the investigators felt it would be more relevant to report all-cause mortality, microbiological failure, and recurrence of MRSA BSI as separate events rather than a composite.

Safety endpoints included (1) nephrotoxicity (defined by an increase in serum creatinine level of 50 μmol/L from baseline or 50% above baseline), (2) musculoskeletal toxicity (defined by a rise in serum CK of five times the upper limit of normal), (3) the need to stop the study drug due to toxicity, (4) the need to discontinue study drug due to worsening infection, and (5) the need for an additional anti-MRSA agent due to worsening infection.

### Assessments

Baseline and serial clinical and laboratory data were collected at specified time points throughout the duration of the study. Blood cultures were performed daily until two consecutive MRSA-negative sets were achieved. Blood tests for full blood count (FBC), creatinine and serum CK were taken weekly. A vancomycin trough level was also measured pre the third or fourth dose of vancomycin and then weekly in patients in the vancomycin arm. An echocardiogram was performed within the first 10 days of randomization to look for evidence of infective endocarditis. All participants had a follow-up assessment 60 days after the index culture, to determine mortality status. A repeat blood culture test was also performed at the last visit.

### Sample size

Simon’s randomized selection design was used to calculate the sample size [17]. A total of 21 participants per arm were needed so as to guarantee a 90% probability of correctly selecting the daptomycin arm as superior to the vancomycin arm if it was truly superior by a margin of 15%. This was calculated assuming a survival rate of 75% at 60 days post index blood culture in the vancomycin arm compared to a survival rate of 90% in the daptomycin arm, based on previously published retrospective case–control and cohort studies [7–9]. This type of design is to select one of two arms as being worthy of further evaluation in a subsequent study but not to confirm the superiority of the selected arm. Assuming an attrition rate of 20%, the target recruitment was a minimum of 50 patients over the course of 2 years.

### Randomization

Once written informed consent was obtained from all patients or their legally acceptable representatives, patients were randomized in a 1:1 ratio using permuted block randomization stratified by site, with the use of a computer-generated list of random numbers. The randomization list was generated by the Singapore Clinical Research Institute (SCRI), with authorized personnel randomizing patients via a direct web randomization system.

### Statistical analysis

A detailed statistical analysis plan was prepared. However due to slow accrual of participants, the Trial Steering Committee took the decision to terminate the study on 19 November 2015 before the target sample size could be reached. Given the small number of patients recruited into the study only descriptive analysis was appropriate. Demographic characteristics and other baseline characteristics (such as clinical measures taken at baseline) were summarized using descriptive statistics (medians and interquartile ranges (IQR) or frequencies (expressed as a fraction of the number of subjects), as appropriate) by treatment group in the intention-to-treat (ITT) analysis population. The frequencies of deaths by 60 days post index culture, microbiologic failure, recurrence of MRSA BSI, nephrotoxicity, musculoskeletal toxicity, the need to discontinue study drug due to toxicity and/or worsening infection, the need to add a second anti-MRSA agent due to worsening infection, and the time to microbiological clearance were summarized by treatment group and overall in the ITT analysis population. The ITT analysis population was defined as all randomized patients. The treatment group of patients in the ITT analysis population was the planned treatment group, i.e. according to the randomization list planned prior to the study commencement. The frequencies of total adverse and serious adverse events were calculated. These were calculated for the treated population that was defined as all randomized subjects who had taken at least one dose of study treatment. The treatment group of subjects in the treated population is according to the treatment actually received after the randomization.

## Results

**Fig. 1:**
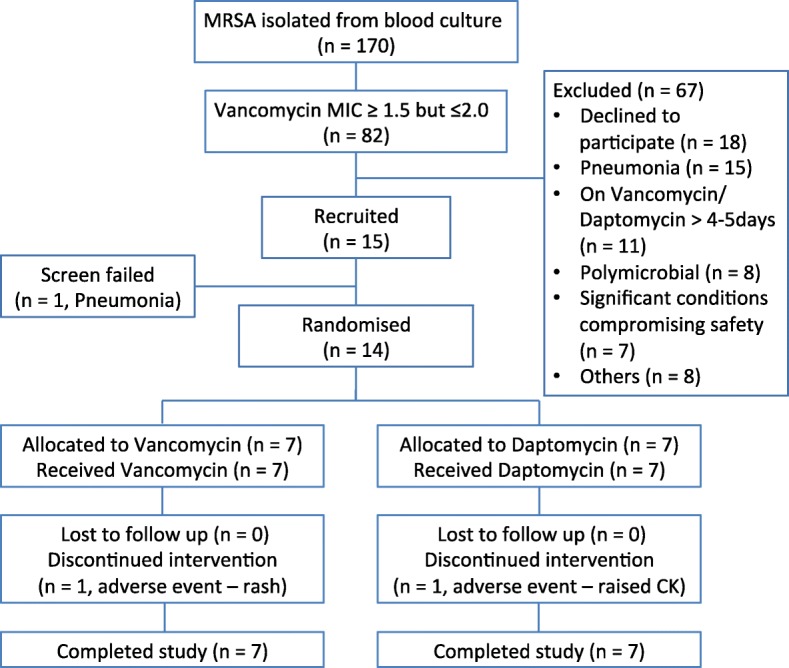
Patient allocation into the study populations. “Significant conditions” included any condition in the investigator’s opinion that would compromise the patient’s safety in the trial. Reasons for exclusion listed under “others” included inability to comply with study treatments and procedures (*n* = 2), inability to provide consent (*n* = 1), prosthetic heart valve in situ (n = 1), creatine kinase (CK) ≥ 1.5 upper limit of normal (*n* = 1), on treatment with linezolid for more than 96 h prior to enrolment (*n* = 1), and patient demise prior to consent (*n* = 2). MRSA, methicillin-resistant Staphyloccus aureus; MIC, minimum inhibitory concentration

A total of 14 patients were randomized in this study. Figure 1 displays the allocation of patients into the study arms. Seven patients were randomized to receive vancomycin and seven to receive daptomycin. All patients received at least one dose of study medication. A total of 9/14 patients were recruited after the study exclusion criteria were amended (as described in “Study Population”): of these 9 patients, 4 would have been previously excluded as they had been on treatment with vancomycin for more than 96 h (but for less than 5 days), prior to randomization.

Baseline characteristics of patients in each treatment arm are summarized in Table 1. Almost three quarters (10/14) of the patients were male and had a median age of 67.5 years (IQR 54.5–71 years): 10/14 patients were of Chinese ethnicity, which was consistent with the racial demographic profile of the country where the study was conducted. There were more patients in the vancomycin group with chronic kidney disease (3/7 compared to 2/7) or both diabetes and chronic kidney disease (3/7 compared to 2/7). Most (12/14) of the patients were diagnosed with uncomplicated MRSA bacteremia at the start of the study treatment. The majority (12/14) of patients in both groups received systemic antimicrobial therapy with vancomycin prior to initiation of the study drug. The median duration of vancomycin treatment prior to enrollment was 3.5 days (IQR 3–5 days).

**Table 1:** Comparison of baseline characteristics between both treatment arms

	Vancomycin (*n* = 7)	Daptomycin (*n* = 7)	All (*n* = 14)
Age in years (median, IQR)	70 (64, 71)	65 (50, 71)	67.5 (54.5, 71)
Age > 65 years	4	3	7
Gender			
Male	5	5	10
Female	2	2	4
Ethnicity			
Chinese	4	6	10
Malay	1	0	1
Indian	1	1	2
Others	1	0	1
Charlson comorbidity score (median, IQR)	5 (4, 7)	5 (4, 8)	5 (4, 7)
Diabetes mellitus prior to screening	0	2	2
CKD prior to screening	3	2	5
Diabetes mellitus and CKD prior to screening	3	2	5
On vancomycin prior to study enrolment	5	7	12
Duration of vancomycin treatment prior to enrolment, days (median, IQR)	3 (3, 5)	4 (3, 5)	3.5 (3, 5)
On daptomycin prior to study enrolment	0	0	0
Diagnosis at enrolment			
Uncomplicated bacteremia	7	5	12
Complicated bacteremia without endocarditis	0	2	2
Endocarditis	0	0	0
Vancomycin MIC (median, IQR)	1.5 (1.5, 1.5)	1.5 (1.5, 1.5)	1.5 (1.5, 1.5)

Data are number of cases, unless otherwise stated

*Abbreviations: CKD* chronic kidney disease, *MIC* minimum inhibitory concentration, *IQR* interquartile range

All baseline isolates of MRSA were susceptible to vancomycin with a MIC of 1.5 μg/ml except for one patient in the vancomycin arm, with a MIC of 2 μg/ml. Median effective duration of treatment with the study drug was 15 days (IQR 14–41 days) in the vancomycin arm and 15 days (IQR: 14–27 days) in the daptomycin arm. There were no deaths in the daptomycin arm and one death in the vancomycin arm. Overall median time to microbiological clearance was 4 days (IQR 3–5 days) in the vancomycin arm versus 4 days (IQR 3–7 days) in the daptomycin arm.

There were no cases of recurrence of MRSA BSI in the daptomycin treatment arm and only one case in the vancomycin treatment arm. This was a 70-year-old woman with end-stage renal failure who was on hemodialysis. She had a tunneled dialysis vascular catheter in-situ, which was removed immediately upon diagnosis of MRSA BSI. She achieved microbiological clearance within 4 days and a new dialysis catheter was inserted. She completed a total duration of 14 days of vancomycin for uncomplicated bacteremia. However, 18 days after completion of antibiotic therapy she developed another MRSA BSI The vancomycin MIC of 1.5 μg/ml remained unchanged. The patient was treated with daptomycin but developed a maculopapular rash and subsequently completed the rest of her treatment with linezolid. Her day-60 blood culture test was negative.

There were no cases of microbiological failure in either treatment arm. One of the seven patients in the vancomycin arm did not complete the study treatment due to development of a vancomycin allergy, which manifested as maculopapular rash. One of the seven patients in the daptomycin arm did not complete the study treatment due to musculoskeletal toxicity with markedly elevated CK. None of the patients in either treatment arm required cessation of study treatment or addition of a second anti-MRSA agent because of worsening infection. One of the seven patients in the daptomycin arm experienced musculoskeletal toxicity, compared to none in the vancomycin arm. None of the patients in the vancomycin arm experienced drug-related nephrotoxicity compared to one patient in the daptomycin arm. The primary and secondary efficacy outcomes of the study are summarized in Table 2.

**Table 2:** Primary and secondary efficacy and safety outcomes

Study variables	Vancomycin (*n* = 7)	Daptomycin (*n* = 7)	All (*n* = 14)
Day-60 mortality	1	0	1
Microbiological failure	0	0	0
Recurrence	1	0	1
Time to microbiological clearance, days (median, IQR)	4 (3, 5)	4 (3, 7)	4 (3, 5)
Musculoskeletal toxicity	0	1	1
Nephrotoxicity	0	1	1
Study drug discontinued due to toxicity	1	1	2
Study drug discontinued due to worsening infection	0	0	0
Addition of a second MRSA agent due to worsening infection	0	0	0

Data are number of cases, unless otherwise stated

*Abbreviations: IQR* interquartile range, *MRSA* methicillin-resistant staphylococcus bacteremia

Overall, adverse events (AEs) were similar in the two study arms (Table 3). Of note, 3/7 patients in the vancomycin arm had dermatologic manifestations, compared to none in the daptomycin arm. These dermatologic manifestations included pruritus and maculopapular rash.

**Table 3:** Adverse events in the treated population

	Vancomycin (*n* = 7)	Daptomycin (*n* = 7)	All (*n* = 14)
Serious adverse events^a^	4	3	7
Adverse events			
Cardiac arrhythmia	2	1	3
General cardiac^b^	2	1	3
Constitutional symptoms^c^	0	2	2
Dermatologic^d^	3	0	3
Adrenal insufficiency	0	1	1
Eosinophilia	0	1	1
Fall	1	1	2
Nausea and vomiting	0	1	1
Anemia	1	1	2
Infection^e^	3	4	7
Neurological^f^	2	1	3
Pain	2	1	3
Hematuria	0	1	1

Data are number of cases

^a^In the vancomycin arm this included intracranial hemorrhage, catheter-related infection, acute coronary syndrome and vascular access complication. In the daptomycin arm this included catheter-related infection and hypotension

^b^Including adverse events termed “acute coronary event”, “hypotension”, and “pulmonary edema”

^c^Including events termed “somnolence” and “fever”

^d^Including events termed “rash-maculopapular” and “pruritus”

^e^Including events termed “catheter-related infection” and “urinary tract infection”

^f^Including events termed “seizure”, “intracranial hemorrhage”, and “dizziness”

There was one death in the vancomycin arm and none in the daptomycin arm. The patient who died was a 71-year-old man with an MRSA BSI due to a urinary tract infection and upper limb cellulitis. His other comorbidities included ischemic heart disease, sick sinus syndrome and severe chronic obstructive pulmonary disease requiring long-term oxygen therapy. On day 2 of study treatment, the patient suffered a cardiac arrest. Prior to his demise, the patient had achieved microbiological clearance. The coroner determined the cause of death as ischemic heart disease.

## Discussion

A rise in MIC to vancomycin within the susceptible range (≤ 2 mg/L) has been observed over recent years [18–20]. This phenomenon has been termed “MIC creep”. Patients with MRSA BSI with a vancomycin MIC ≥ 1.5 μg/ml tend to have higher treatment failure rates (ranging from 50 to 90%) compared to those with lower MIC values [21–25]. A number of studies have also suggested that vancomycin use, especially in infections caused by MRSA with high vancomycin MICs, may be associated with poorer clinical outcomes when compared with an alternative anti-MRSA agent such as daptomycin [7–10].

Fowler and colleagues conducted a prospective open-label randomized controlled trial to compare the success of treatment with daptomycin versus standard treatment with vancomycin plus gentamicin in patients with *S*. *aureus* bacteremia and endocarditis [26]. In subset analysis daptomycin had a greater success rate (although not statistically different) among patients with MRSA BSI when compared to standard treatment (44% with daptomycin vs. 31.8% with standard treatment; *p* = 0.28). However, almost all of the MRSA isolates in this study had an MIC ≤ 1 μg/ml, hence it is difficult to conclude from these results whether daptomycin is truly superior to vancomycin in the treatment of MRSA infections with high vancomycin MICs. Four retrospective studies comparing daptomycin and vancomycin for BSIs due to MRSA with a high vancomycin MIC demonstrated that daptomycin was associated with a more favorable outcome in terms of both clinical success and mortality, compared to vancomycin [7–10]. However, these were retrospective studies so the results need to be interpreted with caution.

In our randomized study, there was only one death and one case of recurrence of MRSA BSI in the vancomycin arm and none in the daptomycin arm. There were no cases of microbiological failure in either treatment arm. Overall outcomes in both arms were better than previously reported in MRSA BSI with a high vancomycin MIC. Earlier studies, which have used varying definitions, have reported a success rate of 61to 73% in patients with MRSA BSI with high vancomycin MICs treated with either vancomycin or daptomycin [7, 8]. There are a number of possible factors that may have led to this improved observed outcome. In our study, vancomycin trough levels were measured at strict intervals to ensure levels were maintained within the therapeutic window of 15–20 mg/ml. In patients whose trough levels were below target, dose adjustments were made in consultation with an infectious disease pharmacist. As such, vancomcyin under-dosing, which has been associated with poorer outcomes [13], was avoided. Patients with concomitant pneumonia, prosthetic valve endocarditis and those with less than 24 h of life expectancy were excluded from our study. These are all conditions that have been associated with poorer overall outcome [27, 28]. In addition, all patients were managed by an infectious disease physician - at least two studies have shown that this is associated with improved outcomes in patients with MRSA BSI [29, 30]. However, due to the small sample size we acknowledge that the improved overall outcome observed may have merely been due to chance.

One patient in the daptomycin arm developed elevated CK compared to none in the vancomycin arm (Table 3). This is consistent with previous studies where CK elevations were more common in the daptomycin group [7, 9, 26]. In our study, three patients in the vancomycin treatment arm had dermatologic manifestations. This is not unexpected, as cutaneous adverse effects have been reported to be the commonest adverse events related to vancomycin use [31–33]. Nephrotoxicity is often a concern with vancomycin, especially in patients with underlying renal insufficiency. However, none of the patients in the vancomycin arm developed significant increases in serum creatinine despite the majority having chronic kidney disease. In our study, vancomycin trough levels were monitored regularly and maintained within a tight therapeutic window of 15–20 μg/ml. In addition, none of the patients received concomitant treatment with an aminoglycoside, which has been shown to increase rates of nephrotoxicity [34]. In contrast, one patient in the daptomycin arm experienced nephrotoxicity, which was deemed by the study investigators to be “possibly related” to daptomycin. To our knowledge, daptomycin induced nephrotoxicity has not been previously reported. This patient was concomitantly receiving high-dose diuretics, which may have led to dehydration and pre-renal impairment. In addition, the patient’s serum creatinine returned to baseline without cessation of study drug. Hence, it remains unclear if the patient’s renal impairment was truly caused by daptomycin.

This study was initially designed as a multicenter study involving three sites. Although 224 patients were prescreened in the three institutions, all 14 patients were eventually recruited from a single institution. None of the 54 patients with MRSA BSI prescreened from the other two institutions were eligible for the study - 52 patients had MRSA BSI with vancomycin MIC < 1.5 μg/ml, one patient had polymicrobial bacteremia and one patient died before he could be recruited. Of the 170 patients prescreened from the eventual recruiting institution, close to 50% (*n* = 82) had MRSA BSI with vancomycin MIC ≥ 1.5 μg/ml but eventually only 14 patients were randomized. The most common reasons for exclusion were concomitant pneumonia in 18% (*n* = 15), exceeding the time allowed on vancomycin prior to enrolment in 13% (*n* = 11) and polymicrobial bacteremia in 10% (*n* = 8). There were 18 patients who were eligible for the study but declined to participate.

The overall recruitment rate of the study was lower than expected with fewer patients than initially projected being eligible for the study. An attempt was made mid-way through the study to improve recruitment by modifying the exclusion criteria (as described earlier) but this did not have a significant impact on improving recruitment rates and the investigators felt that allowing further modifications to the exclusion criteria would adversely affect the safety and integrity of the study. Nine patients were recruited after modification of the exclusion criteria and of these, four patients would have been previously excluded. Of note, 11 patients were excluded because they exceeded the duration allowed on vancomycin prior to enrolment (this was initially capped at 96 h and subsequently increased to 5 days mid-way through the study to try and improve enrolment). As part of our institutional practice, a large proportion of patients are prescribed empirical vancomycin therapy on admission if they have signs of infection and have had recent healthcare contact. Many of these patients would have received more than 4–5 days of vancomycin therapy by the time the vancomycin MIC result was known, making them ineligible for the study. The investigators felt that extending the period allowed on vancomycin therapy beyond 5 days would make it impossible for daptomycin efficacy to be assessed with sufficient rigor. Attempts were also made to increase the number of enrolment sites but these proved unsuccessful, mainly due to the small baseline numbers of patients with MRSA bacteremia at these smaller sites. After consultation with the trial steering committee, a decision was made to terminate the study early as it was felt that it would not be realistically possible to achieve the target sample size within the allocated funding period.

## Conclusions

In summary, based on current available evidence, it remains unclear if alternative, newer and more expensive agents such as daptomycin are truly superior to vancomycin in the treatment of BSI due to MRSA with high vancomycin MIC. The question of what should be the optimal recommended treatment for such infections remains important to answer, and more studies are urgently needed. However, randomized controlled trials evaluating this are complicated and difficult to execute - primarily due to the heterogeneous and complex patient profile associated with MRSA BSI. Enrolment rates are likely to be low and multicenter trials providing a large patient pool will be needed to achieve sufficient patient recruitment for an adequately powered study. An alternative to such conventional randomized controlled trials may be pragmatic adaptive platform trials - such novel methodologies are already being employed successfully in oncology trials [35–37]. Platform studies make use of a universal trial master protocol to study the optimal treatment for a disease and its subgroups, while the pragmatic design embeds the trial in routine provision of care and allows for minimal exclusion criteria, in turn improving recruitment rates. In addition, the use of response-adaptive randomization results in greater probability of patients in a particular sub-group being randomized to interventions that are performing better within that sub-group [37, 38]. Thus, better therapies move through the evaluation process faster, resulting in greater trial efficiency. Investigators interested in evaluating the optimal treatment for MRSA bacteremia may wish to consider employing such novel trial methodologies, so as to ensure a greater chance of success.

## Abbreviations

AEs: Adverse events
BSI: Bloodstream infection
CIRB: Centralized Institute Review Board
CK: Creatine kinase
FBC: Full blood count
IQR: Interquartile range
ITT: Intention to treat
MIC: Minimum inhibitory concentration
MRSA: Methicillin-resistant *Staphylococcus aureus*
SCRI: Singapore Clinical Research Institute
